# Cerebral Glioma Grading Using Bayesian Network with Features Extracted from Multiple Modalities of Magnetic Resonance Imaging

**DOI:** 10.1371/journal.pone.0153369

**Published:** 2016-04-14

**Authors:** Jisu Hu, Wenbo Wu, Bin Zhu, Huiting Wang, Renyuan Liu, Xin Zhang, Ming Li, Yongbo Yang, Jing Yan, Fengnan Niu, Chuanshuai Tian, Kun Wang, Haiping Yu, Weibo Chen, Suiren Wan, Yu Sun, Bing Zhang

**Affiliations:** 1 The Laboratory for Medical Electronics, School of Biological Sciences and Medical Engineering, Southeast University, Nanjing, Jiangsu, China; 2 Suzhou Institute of Biomedical Engineering and Technology, Chinese Academy of Sciences, Suzhou, Jiangsu, China; 3 Department of Radiology, The Affiliated Drum Tower Hospital of Nanjing University Medical School, Nanjing, Jiangsu, China; 4 Department of Neurosurgery, The Affiliated Drum Tower Hospital of Nanjing University Medical School, Nanjing, Jiangsu, China; 5 Department of Oncology, The Affiliated Drum Tower Hospital of Nanjing University Medical School, Nanjing, Jiangsu, China; 6 Department of Pathology, The Affiliated Drum Tower Hospital of Nanjing University Medical School, Nanjing, Jiangsu, China; 7 Philips Healthcare, Shanghai, China; University Hospital of Navarra, SPAIN

## Abstract

Many modalities of magnetic resonance imaging (MRI) have been confirmed to be of great diagnostic value in glioma grading. Contrast enhanced T1-weighted imaging allows the recognition of blood-brain barrier breakdown. Perfusion weighted imaging and MR spectroscopic imaging enable the quantitative measurement of perfusion parameters and metabolic alterations respectively. These modalities can potentially improve the grading process in glioma if combined properly. In this study, Bayesian Network, which is a powerful and flexible method for probabilistic analysis under uncertainty, is used to combine features extracted from contrast enhanced T1-weighted imaging, perfusion weighted imaging and MR spectroscopic imaging. The networks were constructed using K2 algorithm along with manual determination and distribution parameters learned using maximum likelihood estimation. The grading performance was evaluated in a leave-one-out analysis, achieving an overall grading accuracy of 92.86% and an area under the curve of 0.9577 in the receiver operating characteristic analysis given all available features observed in the total 56 patients. Results and discussions show that Bayesian Network is promising in combining features from multiple modalities of MRI for improved grading performance.

## Introduction

Glioma is a common type of brain tumors threatening human lives and the therapies and prognoses are highly dependent on tumor grade[1]. As the current ‘gold standard’ for grading brain tumors, histopathologic grading has two major limitations[2]. One is that the biopsy in some cases (e.g., brain stem) is physically impossible due to its invasiveness. The other is that the biopsy can only sample very limited region in a heterogeneous tumor, which may not reflect the true malignancy of the whole tumor.

Alternatively, with the development of magnetic resonance imaging (MRI), many MRI modalities are of great diagnostic value in evaluating the grade of a glioma noninvasively and as a whole. For instance, blood-brain barrier breakdown is often an indicator of higher grade[2], which can be seen in conventional T1-weighted imaging with gadolinium-based contrast agents (T1W+C). In more advanced imaging modalities, functional information can be obtained for tumor diagnosis. Among them, perfusion weighted imaging (PWI) provides a method to quantitatively measure cerebral microvascularization, whose increase is more characteristic in gliomas of higher grade[3]. This technique exploits the magnetic susceptibility effect caused by the quickly injected contrast agent and the drop in the signal curves during the first pass allows perfusion parameter maps to be extracted after post-processing[4, 5]. The *in vivo* brain multi-voxel MR spectroscopy (also named as MR spectroscopic imaging, MRSI) is another promising approach that enables noninvasive observations of metabolite alterations across tumoral and peritumoral areas[6–9]. For cerebral gliomas, increased choline-containing compounds (Cho) indicate the presence and aggressiveness of a tumor, while decreased N-acetyl aspartate (NAA) is often a sign of neuronal damage and loss by tumors’ invasion. Besides, quantities of lipid metabolites generally reflect the extent of tumoral necrosis which is associated with grade, and lactate (Lac) is also related to tumoral activities.

The more available information can help us have a better understanding of gliomas, but it can also complicate the grading process, especially for inexperienced radiologists. Hence, it would be advantageous to provide a method that can automatically combine different types of information in grading. For this goal, Bayesian Network (BN) [10, 11] is potentially a candidate for its power and flexibility that have been confirmed in many applications of medical diagnosis [12–16]. In a BN, every node is regarded as a random variable that can be discrete or continuous, or even have higher dimensions. The BN structure is the delineation of casual relationships between individual variables and can be either learned from data or manually determined[17]. After parameter learning with enough data, which is the process of estimation of the distribution parameters for each node, we are able to make probabilistic inferences on certain nodes given some other nodes observed. In the case of glioma grading, we can predict the grade of a tumor according to the inference results.

In this work, we proposed a grading system for preoperative cerebral gliomas using BN integrating features extracted from T1W+C, PWI and MRSI. The BN structures were learned using K2 algorithm in conjunction with manual determination and the distribution parameters were learned using maximum likelihood estimation (MLE) [10]. The grading performance of each BN was evaluated in a leave-one-out analysis. The contributions of each feature were studied and the effectiveness of feature combination in BN was validated.

## Methods

### Patients

56 patients were enrolled in this study from January 2010 to March 2015, at the Drum Tower Hospital in Nanjing, China. There were 30 histopathologically confirmed high grade gliomas (WHO III~IV) and 26 low grade ones (WHO I~II). Out of them, all underwent T1W+C, 51 had PWI and 26 had MRSI, and only 21 patients had both PWI and MRSI data. The patients’ ages ranged from 20 to 83 years with a mean of 49 years. This study was approved by the Ethics Committee of the Drum Tower Hospital, and written informed consents were obtained from all patients.

### MR examination

All the scans were performed preoperatively using a Philips whole body 3.0-Tesla scanner (Best, the Netherlands) with an 8-channel head coil. Conventional sequences (T1-weighted and T2-weighted) were conducted first, followed in turn by PWI, T1W+C and MRSI.

In PWI, the field-echo echo-planar imaging (FFE-EPI) sequence was applied with TR/TE 9.9/5.9 ms and 3 repetitions, along with the injection of gadolinium-based contrast agent (Gd-DTPA). After that, T1W+C was scanned in axial, coronal and sagittal orientations separately.

Two-dimensional-MRSI (2D-MRSI) data were acquired using Point-RESolved Spectroscopy (PRESS) sequence with automatic shimming and water suppression. Measurement parameters included 2000/144 ms (TR/TE), 25 to 30 phase encoding steps, 8 mm section thickness. The field-of-view (FOV) was adjusted to each patient’s brain anatomy, and the volume-of-interest (VOI) was set to cover all tumoral, peritumoral and contralateral normal regions on typical slices of either T2-weighted or T1W+C images by experienced neuroradiologists.

### Feature extraction

**Fig 1:**
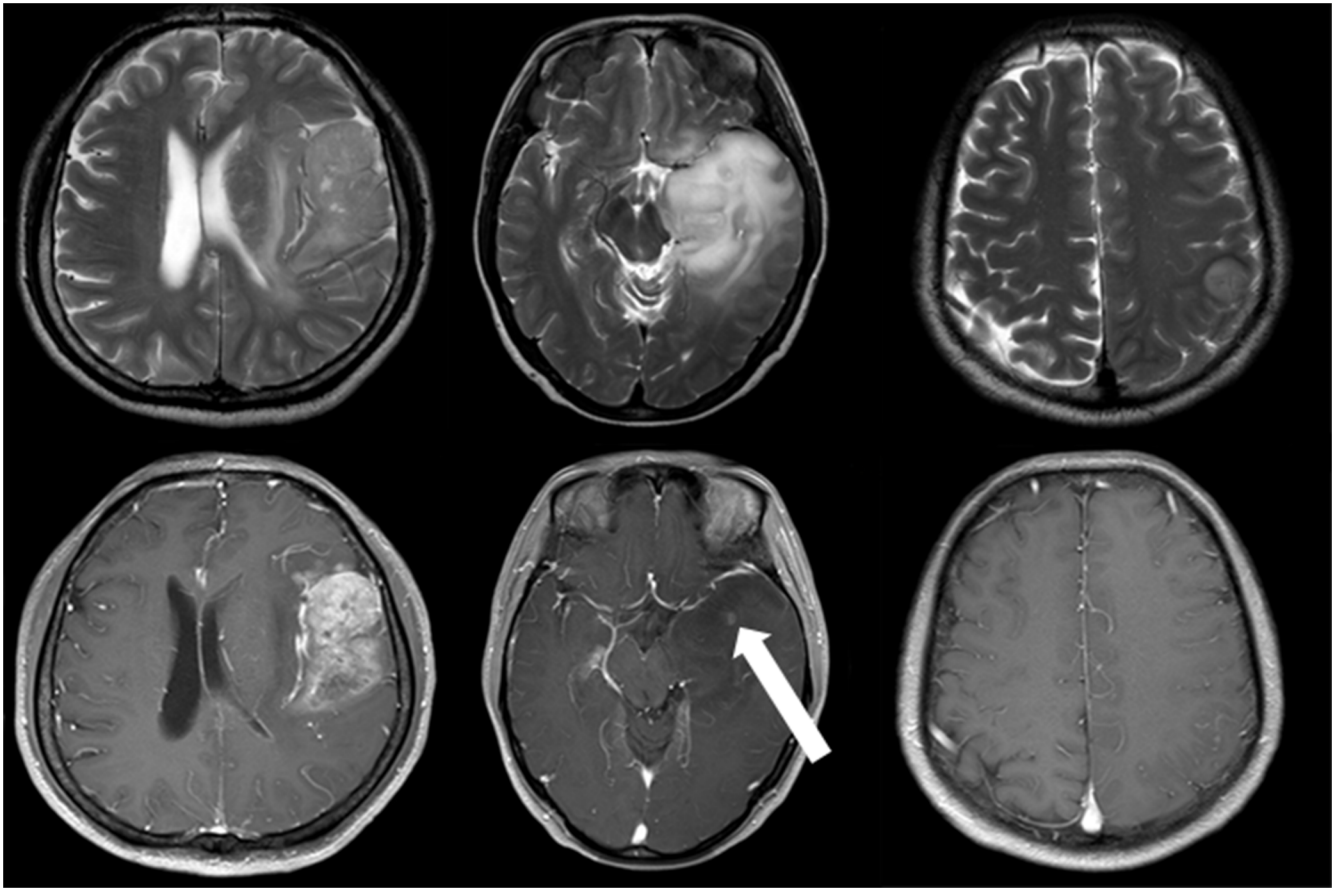
Examples of different enhancement extent. The left column is a case of glioblastoma with apparent enhancement, the middle a case of high-grade glioma with slight enhancement (white arrow) and the right a low-grade glioma with negative enhancement. Upper row axial T2-weighted images. Lower row axial T1W+C images.

The T1W+C feature was extracted according to the enhancement extent of the tumors on the images. This feature was defined to have three states: negative enhancement, slight enhancement and apparent enhancement (Fig 1). Intuitively, negative enhancement means no enhancing areas can be recognized while tumors with apparent enhancement present obvious enhancing regions or highly enhanced signal intensities. As for slight enhancement, there exist some cases where the enhancing areas are small (less than or equal to 5 mm in diameter) and the signal intensities are lower, which can only be observed through careful searching on all axial, sagittal and coronal T1W+C images. Hence, we defined this situation as slight enhancement.

For perfusion features, the PWI data were processed by the Philips workstation and the following parameters were obtained by fitting the signal curves in individual voxels: regional cerebral blood volume (rCBV), mean transit time (MTT), regional cerebral blood flow (rCBF), time of arrival of the contrast agent in the slice after injection (T0) and time corresponding to the maximum contrast variation (time to peak, TTP). Generally, these derived parameters vary a lot among different patients. In order to eliminate this individual difference, two regions-of-interest (ROI) were placed on tumoral and contralateral normal regions respectively, and the parameters from tumoral ROIs were normalized by those from contralateral ROIs. Therefore, the normalized parameters were used as the perfusion features for each case (nrCBV, nMTT, nrCBF, nT0 and nTTP).

**Fig 2:**
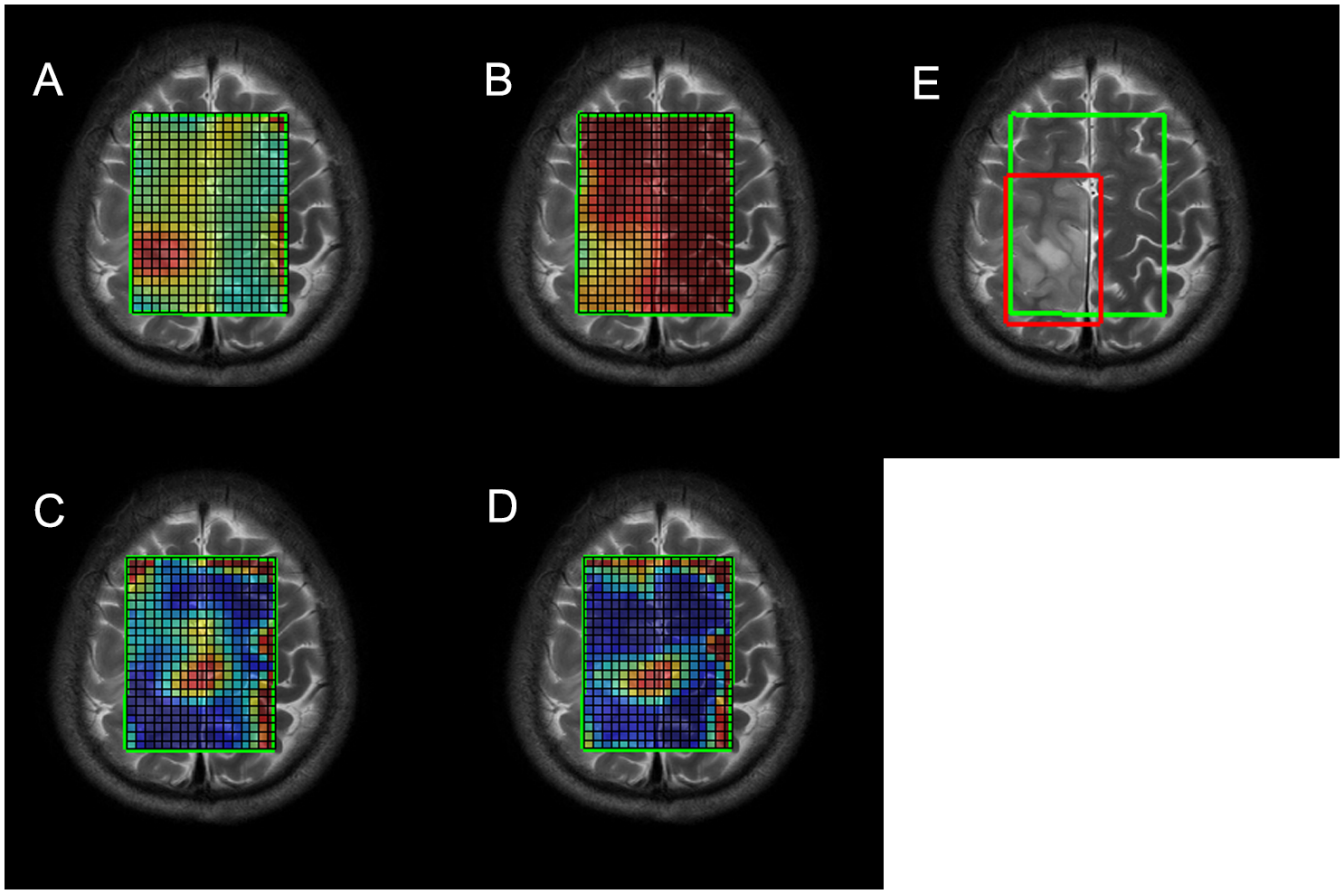
The pseudo-color maps of metabolite ratios in MRSI. (A) Cho/Cr map. (B) NAA/Cr. (C) Lac/Cr map/ (D) Lip13/Cr map. (E) The VOI boundary (green) and the manually selected region (red).

Metabolites in MRSI were first quantitated using LCModel[18, 19] with the processing chemical shift ranging from 4.0 ppm to 1.0 ppm. In LCModel, a spectrum is treated as a linear combination of all the basis metabolites detectable at a certain echo time and the quantitation results are an optimal set of estimated coefficients to fit the spectrum and minimize the residual error. In each voxel within the VOI, all metabolite concentrations were obtained along with their corresponding standard deviations (SD) in percentage, which are measures of confidence in the quantitated concentrations and are associated with the metabolites’ true concentrations and signal-to-noise ratios (SNR) in each voxel. Since the ratios relative to creatine (Cr) are of more clinical importance, the maximums of Cho/Cr, Lac/Cr, Lip13/Cr (lipid at 1.3 ppm) and the minimum of NAA/Cr across the VOI were selected as the representative MRSI features for each case. Considering the low SNRs and shifting baselines in some voxels, some restrictions were imposed on the searching regions. First, a manually defined rectangular region was placed that was guaranteed to just cover the tumoral area (Fig 2E) and the search was confined within the intersect of this region and the VOI. Second, the search was only carried out within the quantitated results with SDs lower than some thresholds. Specifically, the thresholds for Cho/Cr, NAA/Cr, Lac/Cr and Lip13/Cr were set to 20, 60, 50 and 50 respectively. The third restriction was targeted only for Lip13/Cr. Since lipid signals in voxels near the skull and the boundary of VOI will be abnormally high, we limited the search area to be within the voxels that were at least two voxels away from the boundary of the VOI for more reliable Lip13/Cr features (Fig 2D).

### Building Bayesian Network for grading

Prior to network construction, the JMP software was used to perform Kruskal-Wallis test to see if there was statistically significant difference between high grade and low grade groups in the features extracted using the above methods, and the features with P>0.05 were excluded from the construction.

In the method of BN, with constructed networks and learned parameters, the posterior probability of tumor grade was computed given the imaging evidence of a patient and the predictive grade was assigned to the one with the highest probability. In all the constructed networks, tumor grade and T1W+C were defined as discrete random variables, which can take 2 and 3 values respectively, while the rest of the nodes were treated as Gaussian random variables.

With complete data, a typical structure learning algorithm named K2[10, 20] can be used. The K2 algorithm is a greedy search algorithm that works as follows. Initially each node has no parents. It then adds a parent whose addition most increases the score of the resulting structure. When the addition of no single parent can increase the score, it stops adding parents to the node. Considering K2 requires complete and abundant data[10] and yet only 21 patients had both PWI and MRSI data, the network learned using K2 with this very limited dataset might not work well for glioma grading. For this reason, we divided the network into two parts, one for T1W+C and perfusion features and the other for MRSI features, both of which were learned with more available data using K2 (51 for T1W+C and PWI, and 26 for MRSI). For computation efficiency, MLE was used for parameter learning.

The reason of considering T1W+C and perfusion features together is that contrast agent is used in both modalities. We first constructed BNs with different numbers of perfusion features to explore the optimal network for grading, and then T1W+C feature was added to test if it could improve the grading performance. The optimal network for MRSI features was determined with the same method as for perfusion features. In the end, we put together the selected network of T1W+C and perfusion features and the one of MRSI features to validate the usefulness of feature integration by evaluating the grading performance given different combinations of observed features.

Considering the relatively small dataset, the grading performance was evaluated in a leave-one-out strategy. For each test case, the rest of data except the test case were used for parameter learning. The posterior probability of tumor grade was computed given the observed features of this test case, and the predictive grade was checked against the true grade in this case. In doing so for all test cases, the grading accuracy can be obtained by calculating the percentage of the correct predictions.

## Results

### Statistical analysis

Table 1 lists the means, SDs and P values of all the features except T1W+C that can only take discrete values. The P values indicate that all the features except nT0 have statistically significant difference (P<0.05) between low-grade and high-grade gliomas. Hence, nT0 should be excluded from network construction.

**Table 1:** Statistical analysis results of the features.

Features	High grade	Low grade	P value
	Mean + SD	Mean + SD	
T1W+C			<0.0001
nrCBV	3.762 + 2.234	1.482 + 0.624	<0.0001
nMTT	1.483 + 0.779	1.007 + 0.115	0.0007
nrCBF	2.653 + 1.514	1.571 + 0.895	0.0003
nT0	1.048 + 0.212	1.030 + 0.120	0.9773
nTTP	1.088 + 0.168	1.007 + 0.051	0.0014
Cho/Cr	1.169 + 0.522	0.565 + 0.179	0.0005
NAA/Cr	0.357 + 0.186	0.680 + 0.340	0.0044
Lac/Cr	109.598 + 206.765	1.462 + 2.919	0.0001
Lip13/Cr	37.762 + 34.745	1.795 + 2.680	<0.0001

SD, standard deviation; T1W+C, contrast enhanced T1-weighted imaging; nrCBV, normalized regional cerebral blood volume; nMTT, normalized mean transit time; nrCBF, normalized regional cerebral blood flow; nT0, normalized T0; nTTP, normalized time to peak; Cho, choline; NAA, N-acetyl aspartate; Lac, lactate; Lip13, lipid at 1.3 ppm; Cr, creatine.

### Networks of perfusion features

**Fig 3:**
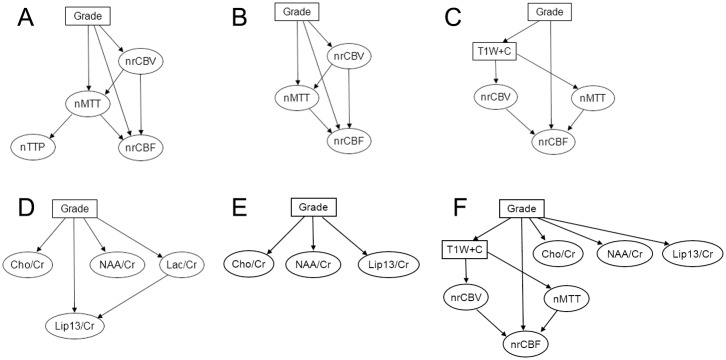
Some BNs constructed in this study. (A) The BN of perfusion features with nTTP. (B) The BN of perfusion features without nTTP. (C) The BN of T1W+C and perfusion features. (D) The BN of MRSI features with Lac/Cr. (E) The BN of MRSI features without Lac/Cr. (F) The final BN structure.

Table 2 presents the grading accuracies achieved by the networks using different combinations of perfusion features. Although the BN with all the perfusion features achieves the highest grading accuracy, the node of nTTP is not directly linked to tumor grade (Fig 3A) and its deletion does not affect the overall accuracy (Fig 3B). This implies that nrCBV, nMTT and nrCBF are the most dominant perfusion features for glioma grading using BN. Form the results of using single features, it can also be observed that nrCBV ranks the first in grading contributions, followed by nMTT and nrCBF.

**Table 2:** Grading accuracies of the BNs with perfusion features.

Features used	Grading accuracy
nrCBV nMTT nrCBF nTTP	0.8431
nrCBV nMTT nrCBF	0.8431
nrCBV nrCBF nTTP	0.8235
nrCBV nrCBF	0.8235
nrCBV nTTP	0.8039
nrCBV	0.8039
nrCBV nMTT nTTP	0.7843
nrCBV nMTT	0.7843
nMTT nrCBF nTTP	0.7059
nMTT nrCBF	0.7059
nrCBF nTTP	0.6863
nrCBF	0.6863
nMTT nTTP	0.6471
nMTT	0.6471
nTTP	0.6275

nrCBV, normalized regional cerebral blood volume; nMTT, normalized mean transit time; nrCBF, normalized regional cerebral blood flow; nT0, normalized T0; nTTP, normalized time to peak.

### Networks of T1W+C and perfusion features

The highest grading accuracy in Table 3 increases to 0.9020 when adding T1W+C to perfusion features (Fig 3C), while both the BNs with either perfusion features or T1W+C feature alone just reach 0.8431. This shows the value of combining T1W+C and perfusion features for glioma grading.

**Table 3:** Grading accuracies of the BNs with T1W+C and perfusion features.

Features used	Grading accuracy
T1W+C nrCBV nMTT nrCBF	0.9020
T1W+C nrCBV nMTT	0.8431
T1W+C nrCBV nrCBF	0.8431
T1W+C nMTT nrCBF	0.8431
T1W+C nrCBV	0.8431
T1W+C nMTT	0.8431
T1W+C nrCBF	0.8431
T1W+C	0.8431

T1W+C, contrast enhanced T1-weighted imaging; nrCBV, normalized regional cerebral blood volume; nMTT, normalized mean transit time; nrCBF, normalized regional cerebral blood flow.

### Networks of MRSI features

In the BNs achieving the highest grading accuracy (Table 4), the node of Lac/Cr is absent in all the networks, which implies that Lac/Cr is not a favorable feature in the current settings of features for glioma grading (Fig 3D). Another point is that using Lip13/Cr alone can achieve the highest accuracy and the contributions of Cho/Cr and NAA/Cr seem not so strong as Lip13/Cr. Nevertheless, considering that Cho/Cr and NAA/Cr are the most frequently measured quantities in clinical diagnosis and the dataset size is limited, we still use the two features. Therefore, Cho/Cr, NAA/Cr and Lip13/Cr are chosen as final MRSI features (Fig 3E).

**Table 4:** Grading accuracies of the BNs with MRSI features.

Features used	Grading accuracy
Cho/Cr NAA/Cr Lip13/Cr	0.8846
Cho/Cr Lip13/Cr	0.8846
NAA/Cr Lip13/Cr	0.8846
Lip13/Cr	0.8846
Cho/Cr NAA/Cr Lac/Cr Lip13/Cr	0.8462
Cho/Cr Lac/Cr Lip13/Cr	0.8462
NAA/Cr Lac/Cr Lip13/Cr	0.8462
Lac/Cr Lip13/Cr	0.8462
Cho/Cr NAA/Cr Lac/Cr	0.8077
Cho/Cr NAA/Cr	0.8077
Cho/Cr Lac/Cr	0.8077
Cho/Cr	0.8077
NAA/Cr Lac/Cr	0.7692
NAA/Cr	0.7692
Lac/Cr	0.7692

Cho, choline; NAA, N-acetyl aspartate; Lac, lactate; Lip13, lipid at 1.3 ppm; Cr, creatine.

### The network of multi-modal features

**Fig 4:**
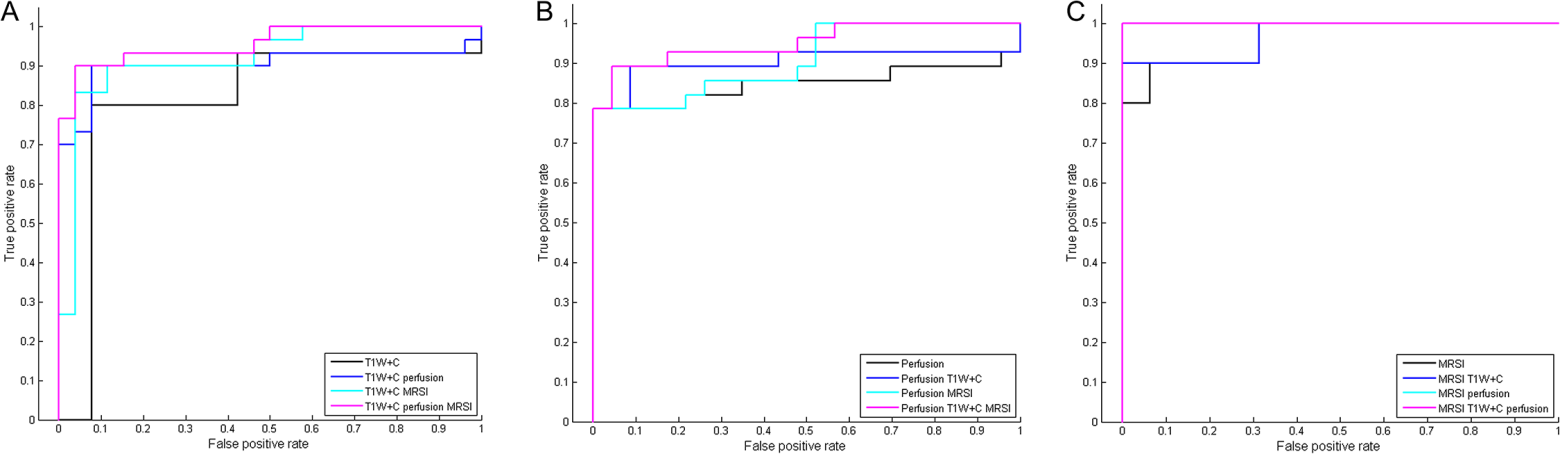
The ROC curves in the three datasets. (A) The ROC curve in the 56 cases. (B) The ROC curve in the 51 cases. (C) The ROC curve in the 26 cases.

Table 5 demonstrates the effectiveness of feature combination in the final constructed BN (Fig 3F) given observations of different modalities. In the total 56 cases with complete T1W+C data, the grading accuracy just reaches 0.8571 when only T1W+C observed, while the added observations of perfusion and/or MRSI features improve the grading accuracies up to 0.9286. In the 51 test cases that all have PWI data, the grading accuracy when only perfusion features observed is below 0.9. When both available T1W+C and MRSI features are observed, this number increases to 0.9216. In the 26 patients with complete MRSI data, the grading accuracy is 0.8846 when only MRSI features observed and it grows to 0.9615 with either additional T1W+C or perfusion features observed. More surprisingly, the supplementary benefit of both T1W+C and perfusion observations makes the accuracy to be 1, meaning predicting all correctly in this dataset. From the receiver operating characteristic (ROC) curves (Fig 4) and the corresponding values of area under the curves (AUC) (Table 5), it can also be noticed that more available observations help improve the grading performance generally.

**Table 5:** Grading accuracies of the final BN with different observations of the features.

Number of test cases	Observed features	Grading accuracy	Number of wrong predictions	AUC
56	T1W+C	0.8571	8	0.8154
	T1W+C perfusion	0.9017	5	0.9038
	T1W+C MRSI	0.8929	6	0.9205
	T1W+C perfusion MRSI	0.9286	4	0.9577
51	Perfusion	0.8824	6	0.8494
	Perfusion T1W+C	0.9020	5	0.9037
	Perfusion MRSI	0.8824	6	0.9099
	Perfusion T1W+C MRSI	0.9216	4	0.9519
26	MRSI	0.8846	3	0.9625
	MRSI T1W+C	0.9615	1	0.9688
	MRSI perfusion	0.9615	1	1
	MRSI T1W+C perfusion	1	0	1

T1W+C, contrast enhanced T1-weighted imaging; MRSI, MR spectroscopic imaging; AUC, area under the curve.

## Discussion

For the aim of more accurate and automatic glioma grading, it is clearly demonstrated that BN is very effective in integrating different features and balancing the contributions among them. Concretely, it is shown that the combination of rCBV, MTT and rCBF performs better than using any one of the three alone, and the added T1W+C feature is further beneficial. Then for MRSI features, results indicate that the most contributing metabolite ratios are Cho/Cr, NAA/Cr and Lip13/Cr. Finally, in the combination of the three modalities, it is also illustrated that the grading performance generally improves as more available features are observed.

In the combination of T1W+C and perfusion features, the highest grading accuracy increases from 0.8431 to 0.9020. The benefit of PWI has been confirmed in many studies. However, a successful PWI acquisition often requires a high speed of injection of the contrast agent [5], while conventional T1W+C images can still be acquired in a traditional way of injection. As a result, some patients may not be well suited for the PWI examination due to their physical conditions. In this situation, the T1W+C feature may still provide evidence of the tumor grade even when PWI data are absent or unusable.

From the evaluation of the final network, it can be seen that more observed features from different modalities generally bring incremental value in predicting glioma grades. In the network structure, apparent enhancement and negative enhancement are strong indicators of high-grade and low-grade gliomas respectively. However, the enhancement extent sometimes fails to reflect the true grade of a glioma [2]. Some low-grade gliomas can still present apparent enhancement and meanwhile no enhancing areas can be seen in some high-grade ones. Moreover, the third defined state of slight enhancement will cause ambiguous predictions when knowing this enhancement extent alone. For these reasons, in the total 56 cases which all have T1W+C features, the grading accuracy just reaches 0.8571 with only T1W+C observed. At a closer look at the 8 misclassified cases, we find that all of them agree with the situations described above. With some additional perfusion and/or MRSI features observed, the grading performance improves accordingly. This shows that perfusion and MRSI features can rectify the wrong predictions by T1W+C feature to some extent in spite of T1W+C feature’s strong influence on grading. In the 51 cases with complete PWI data, the added observations of MRSI features does not help increase the grading accuracy, for all the misclassified cases given only perfusion features observed do not have MRSI data. When perfusion and T1W+C features are observed, there is one misclassified case that has MRSI data and is not in the misclassified ones with only observations of perfusion features. Therefore, further observed MRSI features of this case are responsible for the increase of grading accuracy. In the 26 cases with complete MRSI data, the added observations of T1W+C and/or perfusion features also help to promote the grading performance. Particularly, all predictions are correct with all available features observed in this dataset. From the ROC analysis results in the three datasets, it is also found that using all available observations generally have higher AUC values than using individual features alone, confirming the usefulness of feature combination for glioma grading in BN.

In addition to the benefit of feature combination, BN is a powerful and flexible tool in general and has some other advantages. First, although only two types of distributions are used in this study, the nodes in a BN can be defined to follow different kinds of distributions as needed for certain tasks. Second, the network structures can be learned with certain structure learning algorithms and BN can further serve as a method of feature selection to some degree. Specifically in this study, unlike many earlier publications[3, 21, 22] in which only rCBV or some other single perfusion parameters were used in glioma grading, the usefulness of all five clinically recorded perfusion parameters were investigated by evaluating the grading performance of BNs with different numbers of features. The most likely network structures can be learned using K2 algorithm given the current available complete data. From the relationships of the features in the networks, it turns out that rCBV, MTT and rCBF are the most useful features and TTP can be ignored. Similarly for MRSI features, Lac/Cr is found to have adverse effect to grading accuracies and hence should be removed from the network. More generally, when domain knowledge of data is absent or imperfect, structure learning can help us have a better understanding of the relationships of the nodes in the network and we can find the most contributing features for our task. Note that the final constructed BN is different from the more commonly used method of Naïve Bayes[10, 23] in which all features are conditionally independent of each other given the parent node. The nonlinear behavior of a carefully tuned BN with the necessary links between feature nodes may be more suited for complicated tasks[23]. Third, the ability of dealing with incomplete data is particularly beneficial in the applications like in this study. The usefulness of partial observations has been demonstrated in the previous paragraph. Besides, since the final BN is composed of two parts, where the parameters can be learned with complete data in either part for higher efficiency. In a more general case, the parameter learning can be performed using algorithms like Expectation-Maximization[10] that can make full use of the incomplete data.

Despite the promising use of BN in glioma grading, the major limitation in this work lies in the MRSI dataset, of which both quantity and quality need improvement. On one hand, if we have more available MRSI data, the final BN is likely to make more correct predictions. Further, the network structure can be directly learned with the whole dataset and probabilistic casual relationships between individual MRSI and perfusion features might even exist. For the same reason, only four metabolite ratios are explored in this study. More available data can also make it possible to study the contributions of other metabolites. Nonetheless, the limited MRSI data still show their added value in glioma grading. On the other hand, some MRSI data suffer from low SNRs and distorted baselines, potentially making the quantitation process unreliable. To deal with this problem, some standards should be set for MRSI acquisitions, and quantitation and preprocessing methods can be further refined to generate more effective features[24]. For more general future work, features from other modalities can be added using BN and hopefully detailed grading and classification of brain tumors can be performed with abundant data.

## Conclusion

To aid clinical diagnosis of cerebral gliomas, a grading system based on BN is proposed in this study to integrate features extracted from T1W+C, PWI and MRSI for preoperative cerebral glioma grading. It is demonstrated that feature combination in BN is very effective for improved grading performance even with incomplete data and the general advantages of BN were discussed in detail. For future studies, the grading performance can be further validated with more available data and this method can be extended to other applications.
