# Correction: Cerebral Glioma Grading Using Bayesian Network with Features Extracted from Multiple Modalities of Magnetic Resonance Imaging

**DOI:** 10.1371/journal.pone.0157095

**Published:** 2016-06-02

**Authors:** Jisu Hu, Wenbo Wu, Bin Zhu, Huiting Wang, Renyuan Liu, Xin Zhang, Ming Li, Yongbo Yang, Jing Yan, Fengnan Niu, Chuanshuai Tian, Kun Wang, Haiping Yu, Weibo Chen, Suiren Wan, Yu Sun, Bing Zhang

There is an error in affiliation 1 for authors Jisu Hu, Suiren Wan, and Yu Sun. Affiliation 1 should be: State Key Laboratory of Bioelectronics, Laboratory for Medical Electronics, School of Biological Sciences and Medical Engineering, Southeast University, Nanjing, Jiangsu, China.
